# The Effect of Marital Status on Survival of Patients with Gastrointestinal Stromal Tumors: A SEER Database Analysis

**DOI:** 10.1155/2018/5740823

**Published:** 2018-02-01

**Authors:** Wei Song, Chuan Tian

**Affiliations:** ^1^Department of Intervention and Vascular Surgery, Affiliated Suzhou Hospital of Nanjing Medical University, Suzhou Municipal Hospital, Suzhou Cancer Medical Center, Suzhou, China; ^2^Department of Nuclear Medicine, Guizhou Provincial People's Hospital, Guiyang, China

## Abstract

**Background:**

Marital status has been reported to be a prognostic factor in multiple malignancies. However, its prognostic value on gastrointestinal stromal tumors (GISTs) have not yet been determined. The objective of the present analysis was to assess the effects of marital status on survival in patients with GISTs.

**Methods:**

The Surveillance, Epidemiology, and End Results (SEER) database was used to analyze 6195 patients who were diagnosed with GISTs from 2001 to 2014. We also use Kaplan-Meier analysis and Cox regression to analyze the impact of marital status on cancer-specific survival (CSS).

**Results:**

Patients in the married group had more frequency in white people, more high/moderate grade tumors, and were more likely to receive surgery. Widowed patients had a higher proportion of women, a greater proportion of older patients (>60 years), and more common site of the stomach. Multivariate analysis demonstrated that marital status was an independent prognostic factor for GISTs (*P* < 0.001). Married patients had better CSS than unmarried patients (*P* < 0.001). Subgroup analysis suggested that widowed patients had the lowest CSS compared with all other patients.

**Conclusions:**

Marital status is a prognostic factor for survival in patients with GISTs, and widowed patients are at greater risk of cancer-specific mortality.

## 1. Introduction

Gastrointestinal stromal tumors (GISTs) are the most common mesenchymal neoplasms arising from the gastrointestinal (GI) tract and account for 1-2% of all GI tumors [1]. They can occur anywhere along the alimentary tract, most commonly in the stomach with a frequency of approximately 60–70% [2]. They are thought to arise from the interstitial cells of Cajal (ICC), the pacemaker cells of the GI tract [3]. Radical resection with negative microscopic margins (R0) is the most effective therapy for the majority of patients [4, 5]. Nevertheless, the postoperative recurrence rate for patients with localized GISTs can be 50% [4, 6]. Presently, many tumor-specific parameters such as size, location, mitotic index, nuclear pleomorphism, and tumor necrosis are identified as prognostic factors for GISTs [5, 7–10]. However, only tumor size and mitotic index are the most widely used factors to predict the malignant potential of GISTs [11, 12]. Therefore, it is vital to identify potential prognostic factors that predict prognosis and help clinicians implement better therapeutic strategies.

Several studies have suggested that marital status might serve as a promising prognostic factor for survival in multiple cancers [13–15]. It has been suggested that married individuals have longer overall and cancer-specific survival (CSS) than unmarried patients [16–18]. In a large study of multiple cancer sites, married patients were more likely to present with early-stage disease and more likely to receive definitive treatment than unmarried patients [19]. Being married has been shown to be positively associated with overall and cancer-related survival in multiple malignancies, such as hepatocellular carcinoma [15], gastric cancer [20], colorectal cancer [21], and pancreatic neuroendocrine tumors [22]. According to a larger population-based research on information from the SEER database, it is confirmed that unmarried individuals are at a higher risk of suffering from metastatic cancer, undertreatment, and even death causing from their cancer [19]. Nevertheless, the prognostic role of marital status in GISTs has not yet been assessed. Therefore, the primary objective of this study was to assess the prognostic effect of marital status in patients with GISTs. In the present study, we aimed to investigate the effect of marital status on CSS based on a large population data from the SEER database.

## 2. Materials and Methods

### 2.1. Data Source and Patient Selection

We used the SEER Program of the US National Cancer Institute (NCI) to identify 6195 patients who were diagnosed with GISTs from 2001 to 2014. The SEER Program captures approximately 97% of incident cancers, and the 17 SEER tumor registries encompass approximately 28% of the US population [23]. SEER Program collects information on cancer incidence, prevalence, survival, and mortality of patients with cancer.

Patients with GISTs were identified by the cancer staging scheme, version 0204 and histologic code (International Classification of Diseases for Oncology, Third Edition [ICD-O-3], code 8936). Patients were excluded if they had an unknown cause of death or survival month, age at diagnosis was less than18 years, and a prior malignancy had been diagnosed.

This study was based on public data from the SEER database; we obtained permission to access research data files with the reference number 10091-Nov 2016. The data did not include the use of human subjects or personal identifying information. Thus, no informed consent was required for this part of the study.

### 2.2. Study Variables

The cohort was stratified based on marital status at the time of GIST diagnosis, with discrete strata for married and unmarried (widowed, single, separated, and divorced). Individuals in never married and unmarried or domestic partner were clustered together as a single group. Analyses were controlled for several patient variables, including demographics (sex, age, and race), tumor site (stomach, small intestine, rectum, colon, and others), tumor size (≤2 cm, 2–5 cm, 5–10 cm, >10 cm, and unknown), SEER Stage (localized, regional, distant, and unknown), tumor grade (well-differentiated, moderately differentiated, poorly differentiated, undifferentiated, and not differentiated/unknown), treatment (surgery, no surgery, and unknown), and marital status. The primary outcomes of interest in this study were 5-year CSS, which was calculated from the date of diagnosis to the date of cancer-specific death. Deaths attributed to GISTs were treated as events, while deaths from other causes were treated as censored observations.

### 2.3. Statistical Analyses

Baseline clinicopathological characteristics were analyzed with the chi-square test for categorical variables. Survival function estimation was performed with the Kaplan-Meier method and the resulting curves compared with the log-rank test. The hazard ratio (HR) for relationships between each variable and mortality was calculated using Cox proportional hazards multivariable regression. All *P* values were two-sided, and *P* values < 0.05 were considered statistically significant. All statistical analyses were computed using SPSS version 23 (IBM Corporation, Armonk, NY, USA).

## 3. Results

### 3.1. Baseline Patient Characteristics

In totality, 6195 eligible GIST patients were recognized during the 13-year study period (between 2001 and 2014). Of these patients, 3240 were male and 2955 were female. In total, 3787 (61.1%) were married, 2408 (38.9%) were unmarried including 758 (12.2%) widowed, 1074 (17.3%) never married, and 576 (9.4%) divorced/separated. Patients in the married group were more likely to be male (60.0%), while widowed patients have the highest proportion (80.1%) of female patients. Compared with unmarried patients, the married individuals had more frequency in white people, more high/moderate grade tumors, and were more likely to receive surgery. Patients in the widowed group had the higher proportion of elderly patients (>60 years) and more common site of the stomach. However, the proportion of married and widowed patients in localized disease was similar. All comparisons were statistically significant (*P* < 0.001).  Table 1 provides patient demographics and pathological features.

### 3.2. Influence of Marital Status on CSS

**Figure 1:**
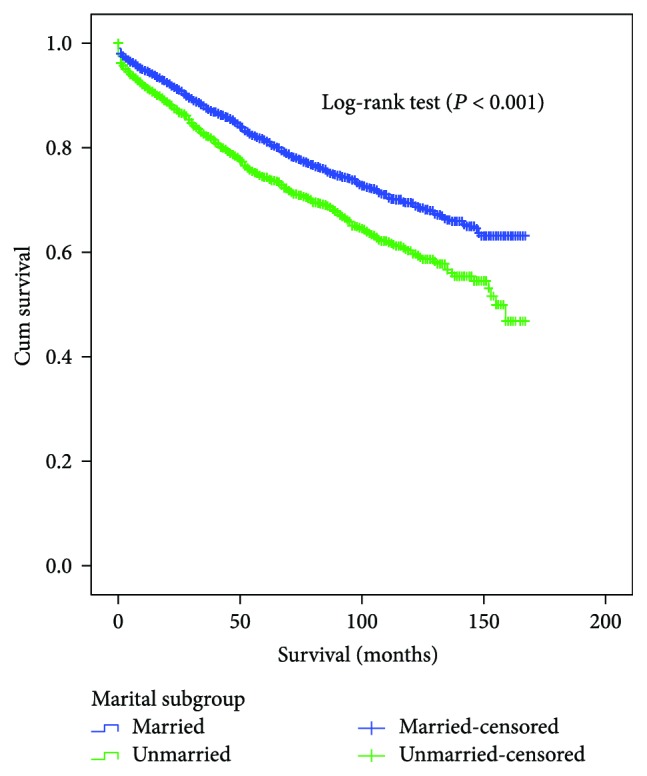
Survival curves in GIST patients between the married patients and the unmarried patients. *χ*^2^ = 41.303, *P* < 0.001.

**Figure 2:**
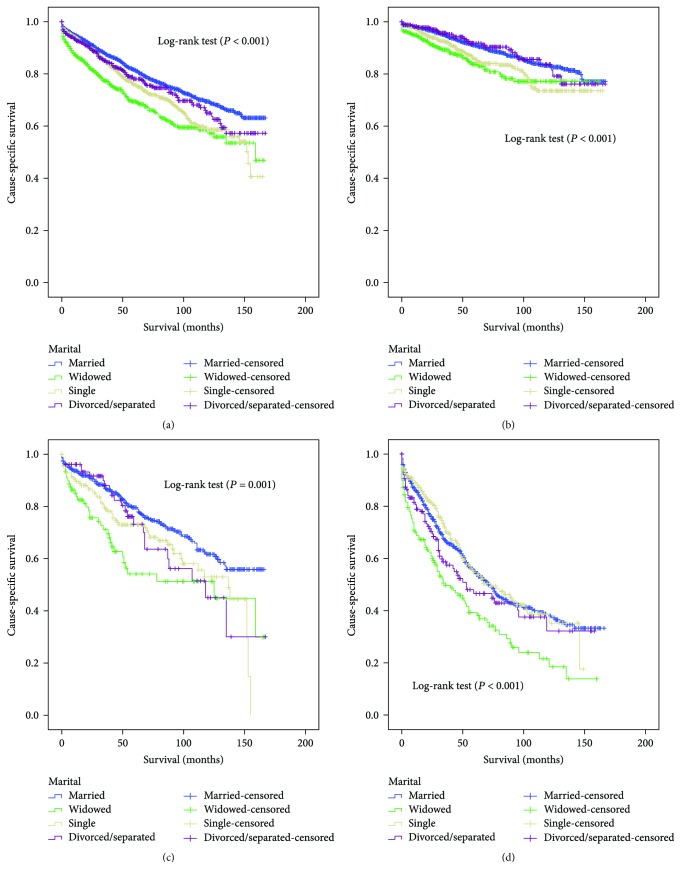
Survival curves in GIST patients according to marital status. (a) All stage: *χ*^2^ = 60.533, *P* < 0.001. (b) Localized: *χ*^2^ = 22,360, *P* < 0.001. (c) Regional: *χ*^2^ = 17.534, *P* = 0.001. (d) Distant: *χ*^2^ = 24.437, *P* < 0.001.

The 5-year CSS was determined by univariate log-rank test. Patients in the married group had better 5-year CSS (81.5%) than patients who were single (75.8%), widowed (69.4%), and divorced/separated (78.1%) (Figures 1 and 2(a)). Additionally, male sex (*P* < 0.001), elderly patients (*P* < 0.001), black ethnicity (*P* = 0.021), colon GIST (*P* < 0.001), tumor size > 10 cm (*P* < 0.001), grade III or IV (*P* < 0.001), advanced SEER stage (*P* < 0.001), and no surgery patients (*P* < 0.001) were regarded as significant risk factors by univariate analysis (Table 2).

The variables which were significant in the univariate log-rank test were validated as independent prognostic factors by multivariate Cox regression analysis. As shown in Table 2, gender (female, hazard ratio (HR) 0.741, 95% confidence interval (CI) 0.658–0.834), age (>60 years, HR 1.649, 95% CI 1.461–1.861), tumor site (colon, HR 1.916, 95% CI 1.344–2.732; others, HR 1.388, 95% CI 1.133–1.700), tumor size (>10 cm, HR 1.752, 95% CI 1.181–2.599; unknown, HR 1.945, 95% CI 1.322–2.861), pathological grading (grade III/IV, HR 2.965, 95% CI 2.310–3.807; unknown, HR 1.547, 95% CI 1.242–1.927), SEER stage (regional, HR 1.983, 95% CI 1.675–2.348; distant, HR 2.952, 95% CI 2.542–3.428; unknown, HR 1.594, 95% CI 1.288–1.973), therapy (no surgery, HR 2.428, 95% CI 2.121–2.779; unknown, HR 4.142, 95% CI 1.703–10.075), and marital status (widowed, HR 1.674, 95%CI 1.411–1.986; single, HR 1.377, 95%CI 1.183–1.602; divorced/separated, HR 1.233, 95% CI 1.014–1.501).

### 3.3. Subgroup Analysis by SEER Stage

We also analyzed the influence of marital status on CSS at each SEER stage. We had some interesting findings. First, marital status was an independent prognostic factor in each tumor stage both in the univariate and multivariate analysis (*P* < 0.001). Second, patients in the widowed group had the lowest survival rate in comparisons at all SEER stages. Compared with married patients, widowed patients had 7.6% reduction in 5-year CSS at localized stage (91.0% versus 83.7%, *P* < 0.001), 25.5% reduction at regional stage (79.6% versus 54.1%, *P* < 0.001), and 15.6% reduction at distant stage (54.9% versus 39.3%, *P* < 0.001). Third, there was no apparent difference between the married and divorced/separated patients in all stage (Table 3, Figures 2(b)–2(d)).

## 4. Discussion

To the best of our knowledge, this is the first study to date which comprehensively investigates the effect of marriage on CSS in GIST patients. Our study showed that married patients have a better CSS and lower mortality than those unmarried patients. In multivariable analyses, the beneficial effect for married patients lasted even after adjusting for sex, age, race, tumor site, tumor size, pathology grade, SEER stage, and therapy. Moreover, patients in the widowed group were more likely to suffer from survival disadvantages than other patients. In addition, subsequent subgroup analysis, based on SEER stage, validated the prognostic value of marital status in GISTs.

One hypothesis for the worse survival in unmarried patients is delayed diagnosis with advanced tumor stage [19, 24, 25]; however, our study showed that the percentages of patients with all SEER stages were comparable among the four subgroups. Moreover, widowed patients have the highest percentage of localized stage (60.4%) and the lowest 5-year CSS (69.4%). Obviously, delayed diagnosis cannot explain the poor prognosis of widowed patients. The exact mechanisms underlying the prognostic impact of marital status in GISTs are unclear. Several biological, psychological, and social theories have been postulated to explain this phenomenon. It is well known that a diagnosis of cancer is psychologically distressing for most patients [26]. Single cancer patients may display more distress, depression, and anxiety than unmarried patients, since there is no spouse that the patient could share the emotional burden and afford sufficient social supports [27, 28]. In addition, marital status may affect adherence to prescribed treatments. Compared with unmarried patients, married patients were more likely to comply with treatment and to seek treatment at more highly recognized centers, all of which may result in better cancer control [29, 30]. Interestingly, we found that the married patients had the highest proportion of surgery (83.5%), while the lowest proportion of surgery (83.5%) is in the widowed group (77.2%). Thus, the hypothesis of undertreatment for widowed patients might be supported by these findings.

Accumulating evidence suggested that the level of physiological stress and depression may affect cancer outcomes via different mechanisms. Decreased psychosocial support and psychological stress result in immune dysfunction and contribute to tumor progression and mortality [31–33]. Moreover, the lack of social support can reduce the activity of natural killer cells [34], and result in disorders of various endocrine hormones, such as cortisol and catecholamines [31, 33]. Chronic stress may promote cortisol secretion [35, 36]. Increased cortisol levels may downregulate the cortisol receptors in white blood cells, thus reducing anti-inflammatory response and promoting cytokine-mediated inflammatory processes [37]. Several studies showed that cortisol and catecholamines could accelerate the growth and metastasis of malignant tumors via immunosuppressive actions [38–40]. Besides, cortisol patterns have been validated as a favorable prognostic factor in breast and lung cancers [36, 40]. Additionally, depression and quality of life are associated with an increased production of VEGF, which may stimulate endothelial cell proliferation, migration, and proteolytic activity [41]. Burgess et al. found that depression and anxiety were associated with breast cancer recurrence [42]. Stress mediators produced in chronic stress could result in tumor metastasis through activation of specific signaling pathways and the tumor microenvironment [33].

Although the present study is based on a large population, some limitations need to be addressed. First, the SEER database only provides the marital status at diagnosis. However, the marital status of some patients may change during the therapeutic process, and these changes may have affected the outcomes. Second, the SEER database lacks details about the duration of the marriage, quality of the marriage, or length of being single, which might influence the prognosis of GIST patients. Marital distress has long-term immune consequences and increases the risk of various health problems [43]. Third, the SEER database did not provide some important information regarding adjuvant therapy, comorbidities, recurrence, or income/insurance status, which could not be adjusted by our analyses.

Despite these limitations, this study was based on a large population and multiple centers and is therefore reliable and persuasive. Our findings demonstrated that marital status is an independent prognostic factor for survival in patients with GISTs. Furthermore, unmarried GIST patients, especially widowed patients, are at greater risk for cancer-specific mortality. The main reasons for poor survival in unmarried patients can be explained hypothetically by social support and psychological factors. Therefore, more social supports should be provided for unmarried patients, especially the widowed patients.

## Figures and Tables

**Table 1:** Baseline demographic and tumor characteristics of patients in SEER database.

	Total	Married	Widowed	Single	Divorced/separated	*P* value
Characteristic	(*n* = 6195)	(*n* = 3787)	(*n* = 758)	(*n* = 1074)	(*n* = 576)	
	*N* (%)	*N* (%)	*N* (%)	*N* (%)	*N* (%)	
Sex						<0.001
Male	3240	2273 (60.0)	151 (19.9)	553 (51.5)	263 (45.7)	
Female	2955	1514 (40.0)	607 (80.1)	521 (48.5)	313 (54.3)	
Age						<0.001
≤60	2777	1710 (45.2)	54 (7.1)	708 (65.9)	305 (53.0)	
>60	3418	2077 (54.8)	704 (92.9)	366 (34.1)	271 (47.0)	
Race						<0.001
White	4237	2738 (72.3)	510 (67.3)	623 (58.0)	366 (63.5)	
Black	1102	460 (12.1)	149 (19.7)	332 (30.9)	161 (28.0)	
Others^∗^	856	589 (15.5)	99 (13.1)	119 (11.0)	49 (8.5)	
Tumor site						<0.001
Stomach	3794	2238 (59.4)	523 (69.0)	662 (61.6)	371 (64.4)	
Small intestine	1858	1199 (31.8)	183 (24.1)	317 (29.5)	159 (27.6)	
Rectum	174	116 (3.1)	14 (1.8)	33 (3.1)	11 (1.9)	
Colon	84	26 (0.7)	11 (1.5)	15 (1.4)	10 (1.7)	
Others	285	186 (4.9)	27 (3.6)	47 (4.4)	25 (4.3)	
Tumor size						0.029
≤2 cm	412	268 (7.1)	47 (6.2)	66 (6.1)	31 (5.4)	
2–5 cm	1354	826 (21.8)	175 (23.1)	222 (20.7)	131 (22.7)	
5–10 cm	1579	980 (25.9)	189 (24.9)	270 (25.1)	140 (24.3)	
>10 cm	1236	740 (19.5)	123 (16.2)	253 (23.6)	120 (20.8)	
Unknown	1614	973 (25.7)	224 (29.6)	263 (24.5)	154 (26.7)	
Grade						0.043
I/II	1209	767 (20.3)	133 (17.5)	199 (18.5)	110 (19.1)	
III/IV	631	410 (10.8)	61 (8.0)	111 (10.3)	49 (8.5)	
Unknown	4355	2610 (68.9)	564 (74.4)	764 (71.1)	417 (72.4)	
SEER stage						0.121
Localized	3692	2306 (60.9)	458 (60.4)	599 (55.8)	329 (57.1)	
Regional	826	499 (13.2)	91 (12.0)	159 (14.8)	77 (13.4)	
Distant	1234	719 (19.0)	149 (19.7)	237 (22.1)	129 (22.4)	
Unknown	443	263 (6.9)	60 (7.9)	79 (7.4)	41 (7.1)	
Therapy						<0.001
Surgery	5055	3164 (83.5)	585 (77.2)	850 (79.1)	456 (79.2)	
No surgery	2256	613 (16.2)	173 (22.8)	223 (20.8)	119 (20.7)	
Unknown	36	10 (0.3)	0 (0)	1 (0.1)	1 (0.2)	

^∗^Others include American Indian/Alaska Native and Asian/Pacific Islander.

**Table 2:** Univariate and multivariate survival analysis for evaluating the influence of marital status on CSS in SEER database.

		Univariate analysis		Multivariate analysis	
Variable	5-year CCS	Log-rank *χ*^2^ test	*P*	HR (95% CI)	*P*
Sex		21.361	<0.001		
Male	76.2%			Reference	
Female	81.7%			0.741 (0.658–0.834)	<0.001
Age		67.660	<0.001		
≤60	83.1%			Reference	
>60	75.1%			1.649 (1.461–1.861)	<0.001
Race		7.697	0.021		
White	79.1%			Reference	
Black	76.6%			0.968 (0.835–1.122)	0.666
Others^∗^	80.0%			0.877 (0.737–1.045)	0.142
Tumor site		80.258	<0.001		
Stomach	79.8%			Reference	
Small intestine	80.1%			1.018 (0.895–1.158)	0.785
Rectum	84.0%			0.841 (0.582–1.215)	0.356
Colon	56.3%			1.916 (1.344–2.732)	<0.001
Others	61.2%			1.388 (1.133–1.700)	0.002
Tumor size		282.519	<0.001		
≤2 cm	90.2%			Reference	
>2–5 cm	92.8%			0.714 (0.466–1.094)	0.122
>5–10 cm	83.5%			1.256 (0.846–1.866)	0.259
>10 cm	70.8%			1.752 (1.181–2.599)	0.005
Unknown	68.7%			1.945 (1.322–2.861)	0.001
Grade		197.472	<0.001		
I/II	91.2%			Reference	
III/IV	62.0%			2.965 (2.310–3.807)	<0.001
Unknown	78.2%			1.547 (1.242–1.927)	<0.001
SEER stage		792.340	<0.001		
Localized	89.4%			Reference	
Regional	75.2%			1.983 (1.675–2.348)	<0.001
Distant	52.0%			2.952 (2.542–3.428)	<0.001
Unknown	76.0%			1.594 (1.288–1.973)	<0.001
Therapy		6161.438	<0.001		
Surgery	84.2%			Reference	
No surgery	52.8%			2.428 (2.121–2.779)	<0.001
Unknown	40.0%			4.142 (1.703–10.075)	0.002
Marital status		60.533	<0.001		
Married	81.5%			Reference	
Widowed	69.4%			1.674 (1.411–1.986)	<0.001
Single	75.8%			1.377 (1.183–1.602)	<0.001
Divorced/separated	78.1%			1.233 (1.014–1.501)	0.036

^∗^Others include American Indian/Alaska Native, Asian/Pacific Islander, and unknown. NI: not included in the multivariate survival analysis.

**Table 3:** Univariate and multivariate analysis of marital status on CSS based on different cancer stage.

		Univariate analysis		Multivariate analysis	
Variable	5-year CCS	Log-rank *χ*^2^ test	*P*	HR (95% CI)	*P*
SEER stage					
Localized		22,360	<0.001		
Marital status					<0.001
Married	91.0%			Reference	
Widowed	83.7%			1.792 (1.347–2.382)	<0.001
Single	85.7%			1.498 (1.150–1.952)	0.003
Divorced/separated	91.8%			0.964 (0.654–1.420)	0.852
Regional		17.534	0.001		
Marital status					0.001
Married	79.6%			Reference	
Widowed	54.1%			2.077 (1.425–3.026)	<0.001
Single	73.0%			1.488 (1.080–2.049)	0.015
Divorced/separated	73.2%			1.362 (0.872–2.127)	0.174
Distant		24.437	<0.001		
Marital status					<0.001
Married	54.9%			Reference	
Widowed	39.3%			1.726 (1.365–2.182)	<0.001
Single	54.7%			0.958 (0.762–1.205)	0.714
Divorced/separated	46.5%			1.216 (0.922–1.605)	0.166
